# IL-6 and Akt are involved in muscular pathogenesis in myasthenia gravis

**DOI:** 10.1186/s40478-014-0179-6

**Published:** 2015-01-15

**Authors:** Marie Maurer, Sylvain Bougoin, Tali Feferman, Mélinée Frenkian, Jacky Bismuth, Vincent Mouly, Geraldine Clairac, Socrates Tzartos, Elie Fadel, Bruno Eymard, Sara Fuchs, Miriam C Souroujon, Sonia Berrih-Aknin

**Affiliations:** Sorbonne Universités, UPMC Univ Paris 06, Myology Research Center UM76, F-75013 Paris, France; INSERM U974, F-75013 Paris, France; CNRS FRE 3617, F-75013 Paris, France; Institut de Myologie, F-75013 Paris, France; Department of Immunology, The Weizmann Institute of Science, Rehovot, Israel; INSERM U735, Centre René Huguenin, Saint Cloud, France; Pasteur Institute, Athens, Greece and Department of Pharmacy, University of Patras, Patras, Greece; Open University of Israel, Raanana, Israel; Centre Chirurgical Marie Lannelongue, Paris-Sud University, Service de Chirurgie Thoracique, Vasculaire et de Transplantation cardio-pulmonaire, Le Plessis-Robinson, France; Laboratoire de recherche chirurgicale and INSERM U999, Le Plessis-Robinson, France; UPMC Univ Paris 06, UMR_S975, Paris, France AP-HP Centre de Référence de pathologie neuromusculaire Paris-Est, Institut de Myologie, GHU Pitié-Salpêtrière, Paris, France; INSERM UMR_S975, CRICM; CNRS UMR 7225, Paris, France; Université Pierre et Marie Curie - Paris VI (U76), INSERM U974 – CNRS-AIM (UMR 7215)- Institut de Myologie, G.H. Pitié-Salpêtrière 47, boulevard de l’Hôpital, F-75 561 Paris, Cedex 13 France

**Keywords:** Myasthenia, Muscle, IL-6, Akt, Anti-acetylcholine receptor antibodies

## Abstract

**Introduction:**

Anti-acetylcholine receptor (AChR) autoantibodies target muscles in spontaneous human myasthenia gravis (MG) and its induced experimental autoimmune model MG (EAMG). The aim of this study was to identify novel functional mechanisms occurring in the muscle pathology of myasthenia.

**Results:**

A transcriptome analysis performed on muscle tissue from MG patients (compared with healthy controls) and from EAMG rats (compared with control rats) revealed a deregulation of genes associated with the Interleukin-6 (IL-6) and Insulin-Like Growth Factor 1 (IGF-1) pathways in both humans and rats. The expression of IL-6 and its receptor IL-6R transcripts was found to be altered in muscles of EAMG rats and mice compared with control animals. In muscle biopsies from MG patients, IL-6 protein level was higher than in control muscles. Using cultures of human muscle cells, we evaluated the effects of anti-AChR antibodies on IL-6 production and on the phosphorylation of Protein Kinase B (PKB/Akt). Most MG sera and some monoclonal anti-AChR antibodies induced a significant increase in IL-6 production by human muscle cells. Furthermore, Akt phosphorylation in response to insulin was decreased in the presence of monoclonal anti-AChR antibodies.

**Conclusions:**

Anti-AChR antibodies alter IL-6 production by muscle cells, suggesting a putative novel functional mechanism of action for the anti-AChR antibodies. IL-6 is a myokine with known effects on signaling pathways such as Akt/mTOR (mammalian Target of Rapamycin). Since Akt plays a key role in multiple cellular processes, the reduced phosphorylation of Akt by the anti-AChR antibodies may have a significant impact on the muscle fatigability observed in MG patients.

**Electronic supplementary material:**

The online version of this article (doi:10.1186/s40478-014-0179-6) contains supplementary material, which is available to authorized users.

## Introduction

Myasthenia gravis (MG) is an organ-specific T cell-mediated autoimmune disease in which autoantibodies against nicotinic acetylcholine receptors (AChR) at the postsynaptic membrane are responsible for a loss of functional AChR and impaired neuromuscular transmission. The immunopathogenic mechanisms that cause a loss of functional AChR include antigenic modulation by anti-AChR antibodies, complement-mediated focal lysis of the postsynaptic membrane, and direct interference with binding of acetylcholine to AChR [1,2]. Experimental autoimmune MG (EAMG) mimics human MG in its clinical and immunopathological manifestations. EAMG induced in rats is the most reliable model for delineating the immunopathological factors and processes involved in MG and for investigating therapeutic strategies for MG [3,4], including treatments aimed at reducing impaired muscle function [5].

Despite the vast body of knowledge accumulated in recent years regarding the underlying immunological mechanisms in MG and EAMG, the molecular mechanisms involved in muscle pathology still remain unclear. Studies of both humans and rats have shown an increased expression of AChR transcripts in the muscles of myasthenic patients or EAMG animals [6-8], suggesting a mechanism of compensation that occurs after the autoimmune attack [9,8]. However, the potential involvement of other muscle genes and pathways has not been investigated.

Microarrays have enabled the identification of specific biomarkers in several autoimmune diseases [10,11]. For example, in type I diabetes, transcriptome studies in patients and Non-Obese Diabetic mouse models revealed similar inflammatory pathways inducible by IL-1β and interferons (IFNs) in the periphery that aided the identification of new biomarkers [10]. In MG, very few studies have used this pan-genomic approach. Our own studies comparing the transcriptome of human normal and pathological thymus yielded a discovery of several novel pathways and pathogenic mechanisms involved in the immune deregulation. We found an inflammatory and anti-viral signature in the thymus of MG patients, as well as a deregulation in immunoglobulin production [12,13]. An abnormal expression of two chemokines, CXCL13 and CCL21, led us to extensively explore these chemokines, revealing their role in the development of thymic germinal centers [14]. A similar analysis in EAMG identified the CXCR3/IP10 pathway deregulated in the lymph nodes of the induced rat model [15]. A specific study of this gene family showed that CXCR3 and IP10 were overexpressed in both EAMG and human MG [15]. Finally, anti-CXCR3 molecules are able to prevent the development of MG disease in the rat-induced model [16]. This example illustrates how a molecule discovered in a pan-genomic study could finally be a therapeutic target.

Our goal here is to identify genes and molecular pathways deregulated in the muscles of MG patients and EAMG. To this end, we compared, for the first time, the muscle transcriptome in seropositive MG (SPMG) patients with healthy muscle, and in parallel, we performed a similar analysis in myasthenic rats. Our analyses revealed the involvement of the IL-6 and IGF-1 signaling pathways. Cell culture experiments demonstrated that anti-AChR antibodies increased IL-6. An analysis of Akt, a common molecule downstream of these two pathways, revealed that monoclonal anti-AChR antibodies decreased the phosphorylation of Akt by insulin. In conclusion, these results show that the pathological mechanisms occurring in the muscle of MG patients and EAMG rats are essentially similar and induce profound cellular changes, including deregulation of the IL-6 and IGF-1 pathways.

## Materials and methods

### Study subjects

Biopsies from the pectoralis of MG patients were collected during thymectomies (Marie Lannelongue Hospital, Le Plessis Robinson). RNA was extracted, and the quality controlled as described below. When the RNA was not high quality, it was excluded. Finally, three positive MG patients with common features were included in the microarray experiments. These patients were young (24-, 26-, and 27-year-old) females, positive for anti-AChR antibodies (6, 10, and 27 nM), with a generalized form disease (IIB in 2 cases, and IIIB in 1 case), and untreated by corticosteroids. Since muscle controls from age- and sex-matched individuals were not available at the time of the microarray experiments, a pool of RNA from muscle biopsies from healthy adults (reference HT1008) was provided by Origene Technology (Rockville, MD, USA).

In order to test the IL-6 protein levels in the muscles, another set of muscle biopsies was collected from SPMG patients (12–49 years old; 6 females and 2 males, from Ia–IVb) and control patients (17–51 years old; 5 females and 2 males). The muscle biopsies from MG patients were collected during thymectomy when the patients were in a clinical stable status, and they were rarely very severely affected. Only one patient had a very severe form (IVb). The muscles from the control patients were collected during cardiac surgery, enabling sampling of the same muscle (pectoralis) as in the MG patients. None of the control patients had other skeletal muscle diseases. Thymus histologies from these MG patients ranged from normal (1) to involuted thymus (1) and follicular hyperplasia (4).

For the effects of sera on IL-6 production, 6 SPMG patients (6 females; age range: 18–34 years, 1 with MG severity IIA, 4 with IIB, and 1 with IIIA) and 6 seronegative MG (SNMG) patients (5 females and 1 male; age range: 19–55 years, 3 with MG severity I, 2 with IIB, and 1 with IIIB) were included. SNMG patients were also negative for anti-MuSK antibodies. Patients on corticosteroid treatment were excluded from the study. Sera from 6 healthy controls, aged 18–45 years, were obtained from the French blood bank. Sera from 6 patients suffering from other muscle diseases were also used: 2 with glycogen storage disease, type III, 1 fascio-scapulo-humeral dystrophy, 1 desminopathy, 1 neuroectodermosis, and 1 spinal amyotrophy.

### Ethics and consent

All the procedures were approved by the local ethics committee “Comité de Protection des Personnes (CPP)”, Kremlin-Bicêtre, France (agreement number 06–018). The muscle biopsies were obtained from MG and non-MG patients who signed an informed consent.

### Animals and antigen preparation

Female Lewis rats aged 6–7 weeks were obtained from the Animal Breeding Center of The Weizmann Institute of Science, and were maintained in the Institute’s animal facility. Female C57Bl/6 J mouse aged 5 weeks were obtained from Janvier laboratories and acclimatized one week in the animal facility (University Pierre and Marie Curie) prior to immunization. All of the experiments in this study were performed according to the institutional guidelines for animal care. Torpedo AChR was purified from the electric organ of *Torpedo californica* by affinity chromatography, as previously described [17].

### Induction and clinical evaluation of EAMG

To induce EAMG, rats were immunized once in both hind footpads via a subcutaneous injection of Torpedo AChR (40 μg/rat) emulsified in complete Freund’s adjuvant (CFA) supplemented with additional non-viable *Mycobacterium tuberculosis* H37RA (0.5 mg/rat; Difco Laboratories, Detroit, MI, USA). The control rats were immunized with CFA and H37RA. Clinical signs of EAMG were monitored on alternate days for 8–10 weeks following disease induction, as previously described [15].

Six-week female mice were immunized by subcutaneous injections in both hind footpads and in the back with Torpedo AChR (30 μg/mouse) emulsified in CFA supplemented with H37RA (1 mg/mouse). Control mice were immunized with CFA and H37RA. Approximately 30 days later, the mice received a subcutaneous boost in the back of the same amount of TAChR in CFA, without additional H37RA; the control mice received a similar boost. The mice were monitored for muscle force and weakness every 10 days. A global score based on the animals’ weights, grip force, and ability to remain on an inverted grid was calculated to quantify their clinical state. Each of these three parameters was graded on a scale of 0–3 to yield a final score on 9, where 0 corresponded to healthy mice and 9 corresponded to severely affected mice.

### Microarray experiments

#### Strategy of the microarray

We adopted a strategy previously used for MG thymus analysis using pools of thymic tissues from homogeneous groups of patients [13,18]. Many of the deregulated genes identified by this approach were then validated in biological studies, such as CXCL13 [19], IFNs [12], and CCL21 [14]. By using pools of muscle tissue instead of individual tissue, we focused our analysis on the primary common changes instead of individual changes. This strategy was validated by our biostatistian (GC). Another advantage of using pools is the ability to perform several technical replicates (quadruplicates in the current study), which is impossible with individual tissue given limitations of both tissue and money. Indeed, performing technical replicates is important to strengthen the results since manipulation of a high number of normalized data can lead to a significant rate of false-negative results.

### GeneChip probing and analysis

#### Rat muscle samples

Muscle samples were harvested from rats when they reached a clinical score of 2 [15]. Since the disease is induced in the hind legs, the thigh muscles that are also affected were used for the extraction of total RNA using the RNeasy midi kit (Qiagen GmbH, Hilden, Germany). Two RNA samples were used for each group, and each sample consisted of a pool from three individual rats.

The GeneChip RG-U34A arrays (Affymetrix, Santa Clara, CA, USA) containing probes for 8000 rat genes and 1000 ESTs were used to screen and quantify the mRNA transcript level in rat thigh muscle samples. Probing and analysis of these samples were performed at the Weizmann Institute microarray unit, as previously described in the literature [15]. Genes showing a fold change greater than 2 were selected for further evaluation.

#### Human muscle samples

Total RNA from muscles of MG patients or from muscle controls (Origene Technology) was extracted using the Trizol reagent (Gibco, Paisley, Scotland) and purified, as previously described in the literature [18]. The sample concentration and purity was first assessed using the NanoDrop spectrophotometer. Then the quality control to assess the sample integrity was checked on an Agilent Bioanalyser (Massy, France). For microarray analysis. only high quality RNAs with RIN (RNA integrity number ) higher than 7, in a scale ranging from from 1 (totally degraded RNA) to 10 (completely intact RNA) were used. Twenty μg of total muscle RNA was labeled with cyanine 5 or cyanine 3 using the direct labeling protocol of Agilent optimized for their cDNA chips, as previously described [19]. For each array, the control muscle RNA was crossed with RNA from MG muscle and these comparisons were conducted in quadruplicate. All of the procedures have been detailed elsewhere [19,13]. Briefly, the labeled cDNA was hybridized overnight onto the human 1 cDNA arrays from Agilent (G4100A; 12,814 unique clones) and scanned using a 428 Affimetrix scanner (MWG Biotech). The images were analyzed with a GenePix pro V4.0 (Axon Instruments). The raw data were then corrected by a non-linear transformation (the Lowess algorithm) using a TIGR Microarray Data Analysis System (http://www.tm4.org/midas.html). A statistical tool “Significance analysis of microarrays (SAM)” was used to identify the gene hit lists that were differentially expressed in human muscle MG compared with control muscle [20].

### Expression analysis

The gene hit lists established in parallel in humans and rats were then submitted to two bioinformatic resources, as previously described in the literature [13]. These two resources provide different types of information:

1. GOTree Machine (GOTM) is a web-based platform for interpreting microarray data or other interesting gene sets using Gene Ontology hierarchies (http://bioinfo.vanderbilt.edu/webgestalt/) [21]. Statistical analysis with relatively enriched gene numbers can suggest biological areas that warrant further study. GOTM generates a GOTree, a tree-like structure to navigate the gene ontology directed acyclic graph for input gene sets. GOTM reports enrichments that are statistically significant, as determined by a hypergeometric test.
2. Ingenuity Pathways Analysis identifies pathways that are overrepresented (https://analysis.ingenuity.com/pa Ingenuity Mountain View, CA, USA) [22,23].

### Quantitative real-time PCR

Quantitative real-time PCR (Q-RT-PCR) on rat and mouse samples was performed using a LightCycler (Roche diagnostic) apparatus, as previously described in the literature [15]. Each sample was run in duplicate and the mean values were used for calculations. The expression levels of β-actin and GAPDH were monitored in all rat samples and were found to be similar. The primers were as follows: rat IL-6 forward: 5′-ctagtgcgttatgcctaag-3′, IL-6 reverse: 5′-ccatctggctaggtaaca-3′; rat IL-6R forward: 5′-ctgaatagagatgcccgt-3′, IL-6R reverse: 5′-gtcactcgcgtaaacc-3′; rat GAPDH forward: 5′-ccaaggagtaagaaaccc-3′. GAPDH reverse: 5′-ggtgcagcgaactttat-3′; rat β-actin forward: 5′-tactgccctggctcctagca-3′; β-actin reverse: 5′-tggacagtgaggccaggatag-3; mouse IL-6 forward: 5′-agttgccttcttgggactga-3′; IL-6 reverse: 5′-tccacgatttcccagagaac-3′; mouse IL-6R forward: 5′-agggtgtctgcttcctgcta-3′; IL-6R reverse: 5′-catctgaggccactcagtca-3′; mouse Rpl32 forward: 5′-caccagtcagaccgatatgtgaaaa-3′; Rpl32 reverse: 5′-tgttgtcaatgcctctgggttt-3′.

### Cell culture

For the cellular experiments, we used immortalized cultures of human myoblasts. These cells, called LHCNM2, were previously derived from the pectoralis major muscle of a 41-year-old male Caucasian heart-transplant donor and immortalized by introduction of human telomerase and cyclin-dependent protein kinase 4 [24]. The cells were cultured in medium containing four parts Dulbecco’s modified Eagle’s medium (DMEM; 4.5 mg/ml glucose) and one part medium 199, supplemented with 20% fetal bovine serum. For the differentiation studies, the cells were trypsinized, counted, and 50,000 cells were plated on to a 48-well plate. After 6–7 days, when the cells had become confluent, the proliferation medium was removed and replaced with DMEM (Gibco) supplemented with 10 mg/ml bovine insulin (Sigma-Aldrich, Saint-Quentin Fallavier France) and 100 mg/ml of human apo-transferrin (Sigma-Aldrich), as previously described in the literature [24].

### Cell stimulation

The LHCNM2 cells were plated in 48-well plates (50,000 cells/well). After 24 h, the medium was replaced with a medium containing the MG patient sera (diluted 1/100 in the regular medium) or monoclonal antibodies directed against AChR. Four different antibodies were used: mAb 198 (IgG2a isotype), mAb 35 (IgG1 isotype), mAb 155 (IgG2a isotype) [25], and anti-AChR (IgG1 isotype) from Acris Antibodies GmbH (reference SM1445). The rat IgG2a and IgG1 isotype controls (clones 54447 and 43414) were purchased from R&D Systems, Inc. (Minneapolis, MN). The results were normalized to the control sera or the relevant isotype control, respectively. The monoclonal antibodies and their isotype controls were used at a concentration of 3 μg/ml.

### Cell proliferation

Cell proliferation was assessed by flow cytometry using Carboxyfluorescein Diacetate Succinimidyl Ester (CFSE) dye, as previously described in the literature [26]. Briefly, LHCNM2 cells were treated with CFSE (5 μM/1 × 10^6^ cells) (Molecular Probes, Interchim, France) for 10 min at 37°C. After washings, the cells were seeded in 24-well plates, allowed to attach for 24 h, and then incubated with monoclonal Abs. The cells were then collected after 24, 48, and 72 h incubation and acquired on a FACScalibur (BD Biosciences, Le Pont de Claix, France). The cytometry analysis was completed using Flowjo software Tree Star, Inc. (Ashland, OR, USA).

### ELISA assays

Enzyme-linked immunosorbant assay (ELISA) was performed on culture supernatants, as well as on muscle extracts to measure their IL-6 content. The frozen muscle biopsies from patients were thawed and homogenized in extraction buffer (Tris–HCl pH8 20 mM, NaCl 137 mM, glycerol 10%, NP-40 1%, EDTA 2 mM) supplemented with proteinase inhibitor cocktail (Complete, Roche, France). The homogenates were then centrifuged and the supernatant was kept at −80°C until analysis.

All of the reagents for the ELISA were from Immunotools (Friesoythe, Germany) and used according to the manufacturer’s instructions. Briefly, the plates were coated overnight with the anti-IL-6 antibody at 4°C and washed 5 times. The aspecific sites were blocked for 1 h at 37°C. The plates were washed 5 times and then samples were added, incubated for 1 h at 37°C, and washed 5 more times. The biotinylated IL-6 antibody was added, incubated 1 h at 37°C, and washed 5 times. Streptavidin-HRP was incubated 20 min at room temperature, and washed 5 times. TMB substrate was allowed to incubate for 5 min before adding the stop solution. Optical density (OD) was read at 450 nm in an MRX Revelation ELISA plate reader (Dynex Technologies, Inc. Chantilly, VA, USA). A standard curve was obtained with serial dilutions of IL-6 and the results were expressed in pg/ml. The cytokine concentration was calculating using the mean of the duplicate measurements for each sample.

### Analysis of Akt phosphorylation

Akt phosphorylation was assessed by a Western Blot. The LHCN myotubes and myoblasts were treated for two days with AChR antibodies or control isotype. On the day of the analysis (differentiation day 7 (D7) for the myotubes), they were rinsed and incubated in serum-free medium for 3 h, before being treated with insulin for 10 min. The LHCN cells were rinsed with phosphate buffered saline and the proteins were then extracted with an extraction buffer (Tris HCl pH8 20 mM, NaCl 137 mM, glycerol 10%, NP-40 10%, EDTA 2 mM) supplemented with antiprotease and antiphosphatase mixes (Complete Mini and PhosSTOP, respectively, Roche, France). Cellular debris was eliminated by centrifugation at 14,000 rpm for 20 min at 4°C. The protein extracts were kept at −80°C prior to analysis.

The proteins were thawed on ice, denatured in Laemmli buffer at 95°C for 5 min, separated by polyacrylamide gel electrophoresis (Precise Tris-Hepes gels, Pierce, France), and transferred to a nitrocellulose membrane. The protein transfer was evaluated using Ponceau staining. The membrane was saturated with 5% of milk proteins in Tris buffered saline with 0.05% Tween (TBST), incubated with anti-pAkt (Ser 473) rabbit antibody (dilution 1/500 to 1/1000) (Cell Signaling Technology, Inc., Danvers, MA, USA) overnight at 4°C, washed three times in TBST, incubated for one hour in secondary antibody (anti-rabbit HRP, 1/10000, in 1% of milk in TBST), washed three times in TBST, and revealed with electrochemiluminescence (ECL) Prime on autoradiography films (Amersham, GE Healthcare Bio-Sciences AB, Uppsala, Sweden). The process of immunodetection was repeated with an anti-Akt antibody (Cell Signaling Technology) to assess total Akt. Band intensities were evaluated using Fiji Is Just ImageJ.

### Statistical analysis

For each data set, the normality of the samples was tested using three normality tests (Kolmogorov-Smirnov, D’Agostino and Pearson omnibus, and Shapiro-Wilk). If the samples were characterized by a Gaussian distribution, the significance of the results was analyzed using a Student’s *t*-test or ANOVA (more than 2 groups). Otherwise, nonparametric tests were used: Mann–Whitney for comparison of 2 groups and Kruskal-Wallis non-parametric ANOVA for comparison of 3 groups or more. All analyses were done using GraphPad software (GraphPad, San Diego, CA, USA).

## Results

### Categories of deregulated genes are similar in human MG and rat EAMG muscle arrays

**Figure 1:**
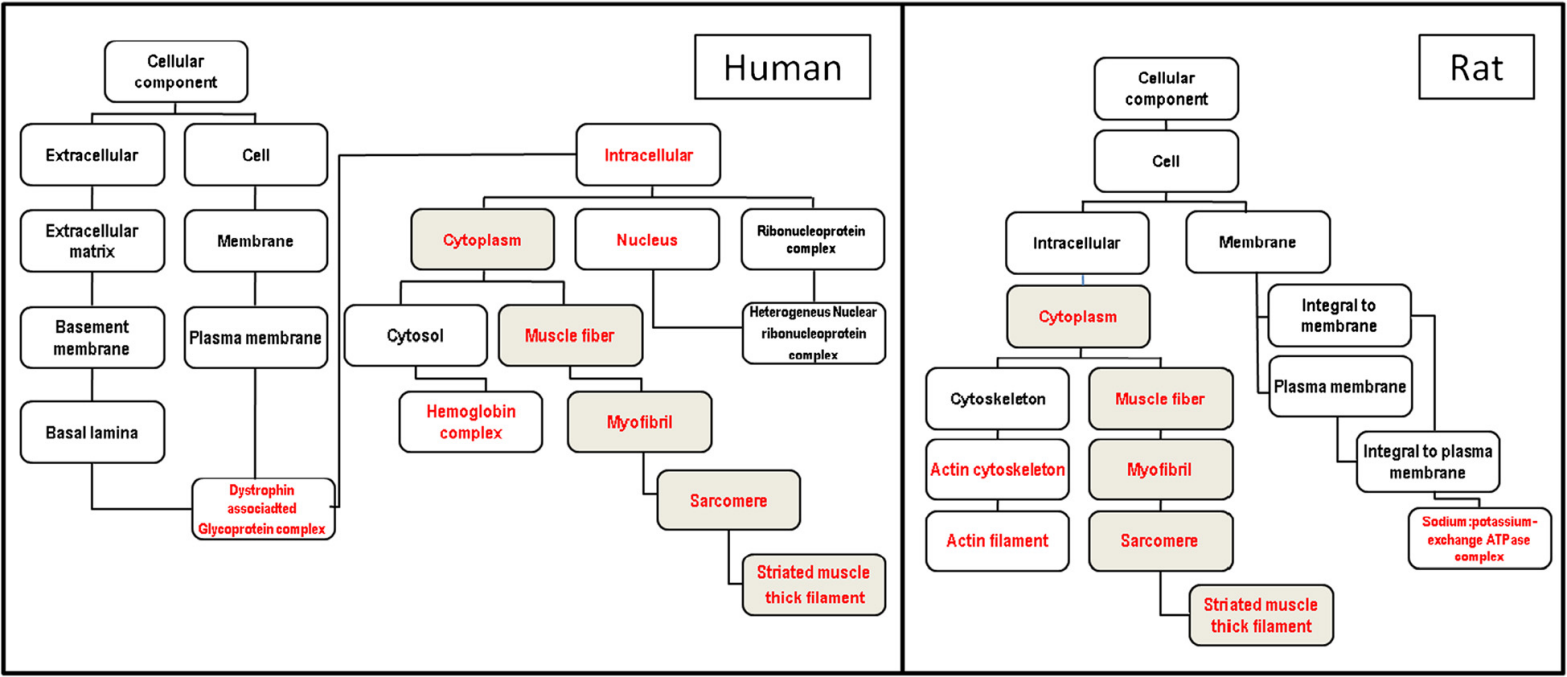
**Analysis of gene categories modified in human and rat myasthenia show similar results.** Scheme of the significantly enriched Gene Ontology categories in the cellular component system. Categories with significantly enriched gene numbers determined by a hypergeometric test (http://bioinfo.vanderbilt.edu/webgestalt/) are indicated in red; categories shown in black are non-enriched. GOTM analysis demonstrated a strikingly similar tree in human and rat muscle and demonstrates the involvement of genes included in the muscle fiber category (grey boxes).

Transcriptome analysis was performed on both data sets, human and rats. The data were analyzed using the GOTM [21] that generates a tree-like structure and yields two types of data: 1) the subcategories presenting statistical changes and 2) the hierarchy between these different subcategories. In this representation, the downstream category is a subgroup of the upstream category. Figure 1 shows a strikingly similar tree for human and rat myasthenic muscle compared with their respective controls and suggests the involvement of genes included in the muscle fiber category in the pathology of MG (deregulated gene categories are marked in red).

### Common signaling pathways are deregulated in MG and EAMG muscles

We used the Ingenuity Pathway analysis software to compare deregulated signaling pathways in the human and rat muscle samples in order to identify cellular pathways that are potentially involved in mechanisms underlying the pathogenesis of MG. In Table 1, we list the major common canonical pathways that are significantly different in both human and rat myasthenic muscles from their respective controls. Interestingly, the IL-6 and IGF1 pathways already known to play a role in muscle metabolism and function [27,28] were significantly represented in the hit lists obtained from microarray analysis of muscle samples from both MG patients and EAMG. The deregulated genes in the IL-6 and IGF1 pathways are listed in Additional file 1: Table S1.

**Table 1:** **Global canonical pathway: human versus rat**

**Canonical pathway**	***Human significance***	***Rat significance***
IGF-1 signaling	6.89*10^−4^	2.53*10^−2^
IL-6 Signaling	1.47*10^−2^	1.12*10^−2^
Nitric oxide signaling in the cardiovascular system	3.39*10^−2^	3.47*10^−2^

Gene expression data for human and rat muscle were evaluated using Ingenuity Pathway Analysis. The analysis used knowledge databases to identify global canonical pathways associated with gene expression. The significance value of a given canonical pathway, a measurement of the likelihood that the pathway is associated with the data set by random chance, was calculated using a right-tailed Fisher’s Exact Test, and values of p < 0.05 were assumed to be statistically significant.

Since IL-6 has already been shown to be associated with pathogenic mechanisms of MG [29], we decided to focus on the IL-6 pathway involvement and the common downstream actor of IL-6 and IGF1 pathways Akt/PKB.

### IL-6 and IL-6R are altered in the muscles of animal models

**Figure 2:**
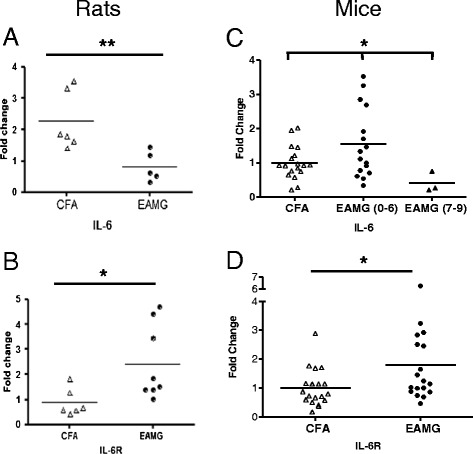
**IL-6 and IL-6R expression is modified in muscle from animal models of MG.** mRNA expression of IL-6 and IL-6R in muscles of EAMG and control CFA-immunized rats **(A** and **B)** and mice **(C** and **D)**. mRNA expression levels of IL-6 **(A** and **C)** and IL-6R **(B** and **D)** were determined by Q-RT-PCR. In IL-6 transcript levels for mice, the EAMG group was separated in EAMG score with a zero-to-moderate clinical score (<6) and EAMG with a high score (>7). The clinical weakness was evaluated by weight loss, reduced grip strength, and reduced ability to hang on an inverted grid. β-Actin for rats and Rpl32 for mice were used as an inner control for normalization of IL-6 and IL-6R. The groups were compared using *t*-tests for significance. ANOVA was used for mice IL-6 transcripts levels (C, p = 0.0315). Post-test comparisons of groups were not individually significant, but a trend could be observed (Controls versus EAMG (0–6) p = 0.0516; EAMG (0–6) versus EAMG (7–9) p = 0.0792) ***p < 0.001; **p < 0.01; *p < 0.05.

By using RT-PCR, we showed that the expression of IL-6 and IL-6R were altered in the muscles of EAMG rats and mice in comparison with control CFA-immunized animals. The expression of IL-6 was 3-fold lower in EAMG rats than in control CFA rats (Figure 2A), but about 2-fold higher in EAMG mice (Figure 2C); the expression of IL-6R was about 1.5–3-fold higher in muscles of EAMG animals than in control-CFA rats or mice (Figures 2B and D). The contradictory IL-6 results between mice and rats may be the result of the disease severity. Indeed, the rat model is more effective and all rats exhibited a severe clinical score, whereas most mice did not show more than a slight muscle strength decrease. Only three mice had a severe phenotype and their IL-6 transcript levels were very low (Figure 2C).

These results suggest that the autoimmune attack on the muscle in the EAMG animal model may trigger the changes in the expression of IL-6 and its receptor.

### IL-6 production is increased in MG muscle and in muscle cells treated with MG sera

**Figure 3:**
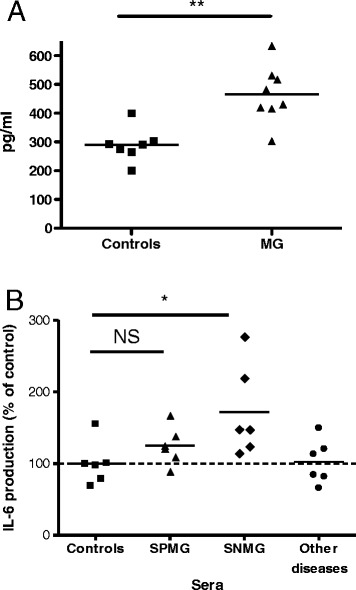
**IL-6 protein is increased in muscle from MG patients and is induced by MG sera in muscle cells.** The IL-6 levels were determined by ELISA in protein extracts of human muscle biopsies **(A)** or in the medium of myoblasts cultured for 24 h with sera from SPMG or SNMG patients, or other skeletal muscle diseases or healthy controls diluted 1/100 in the culture medium **(B)**. Each serum was tested twice. The significance was evaluated using *t*-tests between groups. **p < 0.01; *p < 0.05.

To test whether IL-6 was also modified in human patients, we first analysed the mRNA IL-6 level in MG muscle biopsies. We observed a trend for high IL-6 transcript levels in MG samples compared to controls, but the difference was not significant (data not shown). We then quantified the IL-6 protein in extracts from eight MG muscle biopsies and seven healthy biopsies. The IL-6 protein concentration was significantly higher in muscle extracts from patients compared with healthy controls (Figure 3A). To investigate whether this increased IL-6 production was due to pathogenic effects by MG sera, we tested the effects of sera from MG patients (six SPMG, six SNMG patients, and six age-matched controls) on IL-6 production by human muscle cells. We found that sera from most MG patients (5 out of 6 SPMG and 6 out of 6 SNMG) induced a significant increase in IL-6 production (p < 0.005 for the entire group of MG patients) when normalized to the production by cells treated with sera from age-matched controls. Interestingly, the effect was stronger in the presence of sera from SNMG patients than from SPMG patients. Sera from patients with other muscle diseases had no effect (Figure 3B). The high level of IL-6 in the culture supernatants of cells stimulated with MG sera was due to true production of IL-6 by muscle cells in culture and not to the basal level of IL-6 in the MG sera. Indeed, the IL-6 concentration in the sera ranged from 5–15 pg/ml, and the sera was diluted 100 times. As a result, the contribution of the seric IL-6 in the supernatant was lower than 0.3%.

### IL-6 increase in muscle cells is caused by anti-AChR antibodies

**Figure 4:**
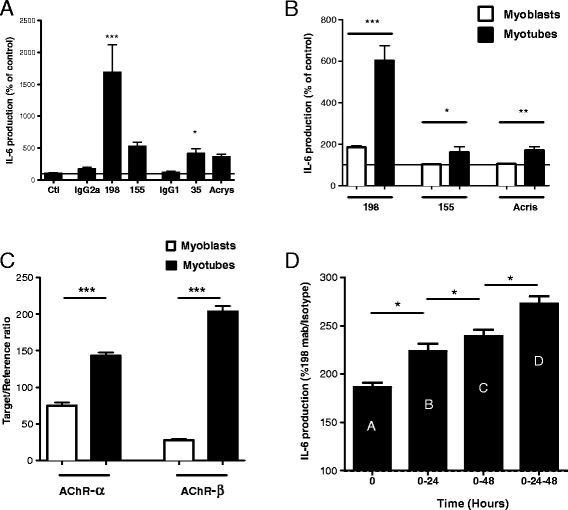
**Monoclonal anti-AChR antibodies increase IL-6 production by muscle cells.** The LHCNM2 cells were cultured as described in the Materials and Methods section, as myoblasts or after differentiation into myotubes. **(A)** IL-6 production was measured by ELISA and normalized to controls (without antibody). Myotubes were treated with anti-AChR antibodies (198, 155, 35 and Acrys), their isotype controls (IgG2a, IgG1), or left untreated (Ctl). The data come from four experiments, each including 4–6 replicates. **(B)** For some antibodies, the effect on myotubes (black bars) and myoblasts (white bars) was compared. The data come from three experiments, each including 2–4 replicates. **(C)** The expression of AChR subunits in myotubes (black bars) and myoblasts (white bars) was assessed by Q-RT-PCR (n = 4). **(D)** Addition of the anti-AChR mAb 198 was performed at several time points and all supernatants were analyzed at 72 h. mAb 198 was added once (bar A), twice (bars B and C), or at three consecutive time points (bar D). The results shown are from two wells for each condition in a representative experiment. Bars and error bars represent mean ± SEM. ***p < 0.001; **p < 0.01; *p < 0.05.

To investigate whether these effects on IL-6 production were due to anti-AChR antibodies, we tested the effect of several anti-AChR monoclonal antibodies (Figure 4). For each antibody, the relevant isotype control was used for normalization (IgG2a or IgG1). MAb 198 and mAb 35 are directed against the main immunogenic region on the extracellular part of the alpha subunit and are known to cause not only antigenic modulation but also passive transfer EAMG [25]. MAb 155 has been described to bind to a major epitope of the cytoplasmic side of the α-subunit [25]. The Acris mAb, a commercial monoclonal antibody, reacts with an unknown epitope of the human AChR. We observed that mAb 198 had a larger effect on IL-6 production by myotubes than mAb 35, mAb 155 or Acris mAb (Figure 4A). These data suggest that the induction of IL-6 by anti-AChR mAbs is dependent on the epitope recognized by the antibody.

We compared the effect of antibodies on undifferentiated myoblasts and myotubes. Interestingly, the IL-6 increase induced by anti-AChR antibodies was higher in myotubes than in myoblasts for all of the antibodies. For mAb 155 and Acris, the IL-6 levels were unchanged in myoblasts (Figure 4B). This finding could be related to the higher expression of AChR in differentiated myotubes, which is illustrated here with the mRNA level of the α- and β- subunits (Figure 4C).

In MG, anti-AChR antibodies are produced continuously. Therefore, in order to better mimic the pathological situation, we added anti-AChR mAb 198 at several time points during cell culture and analyzed the IL-6 production. Figure 4D shows that the system is not saturated, since the addition of anti-AChR antibodies at three successive time points (Figure 4D, bar D) resulted in a higher production of IL-6 compared with additions at a single time point (Figure 4D, bar A) or two time points (Figure 4D, bars B and C).

### Antibody effects on IL-6 production are due to a transcriptional mechanism

**Figure 5:**
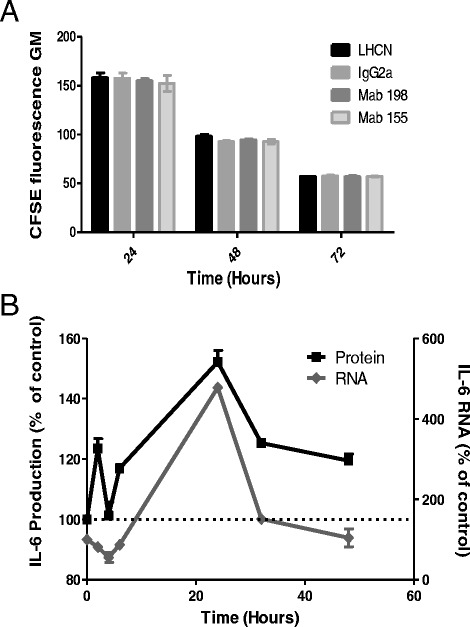
**Mechanisms of action of mAb 198. (A)** Comparison of the proliferation of human muscle cells in the presence of anti-AChR antibodies or isotype control using CFSE. Fluorescence geometric means (GM) decreased with time but did not exhibit any differences when the cells were treated with anti-AChR mAb or isotype control (3 μ/ml). **(B)** Kinetics of IL-6 production by LHCNM2 after stimulation with mAb 198 anti-AChR antibody is represented in black, and mRNA expression is represented in grey. IL-6 production was measured by ELISA assays and normalized to isotype controls (n = 4 for each group and time). mRNA expression was measured by Q-RT-PCR and was normalized to GAPDH (n = 2 for each group and time). Mean ± SEM is represented.

To determine whether the increased IL-6 production was due to an increased cell number induced by anti-AChR antibodies, we compared the proliferation of human muscle cells in the presence of anti-AChR antibodies or isotype controls using CFSE labeling. There was no difference in the CFSE fluorescence intensity whether the cells were cultured in the presence of anti-AChR or isotype-matched antibodies (Figure 5A). These results suggest that the anti-AChR antibodies do not induce muscle cell proliferation, emphasizing that the increased production of IL-6 was not due to an increased number of cells.

To analyze whether the antibody effects on IL-6 production were due to a transcriptional mechanism, we performed a kinetic study on protein and RNA levels in the muscle cells in the presence of mAb 198. The results were normalized to the IgG2a isotype control. As shown in Figure 5B, the mRNA and protein levels followed parallel curves, albeit with higher variations for the mRNA (up to 400% and 150% for mRNA and protein levels, respectively). Interestingly, 2 hours after the addition of antibodies, we observed an early peak of protein expression associated with a marked reduction in the IL-6 mRNA level, suggesting that, at the early phase, IL-6 is translated from a pre-existing pool of IL-6 mRNA, as previously described for AChR expression [8].

Altogether, these results indicate that the effects of anti-AChR antibody on IL-6 production cannot be attributed to a higher proliferation of myoblasts, but rather to an increased transcription rate or a post-transcriptional mechanism such as increased transcript or protein stability. It is noteworthy that the increased level of transcripts was acute and transient while the high IL-6 protein expression was moderate and quite stable.

### Phosphorylation of Akt, a downstream effector of IGF-1 and IL-6, is impaired by AChR monoclonal antibodies

In order to understand how muscle cells are affected, we investigated a downstream effector of IGF-1 and IL-6, PKB/Akt. The Akt pathway plays several roles in muscle physiology. For instance, it promotes growth through protein synthesis and mediates glucose capture through translocation of glucose transporter (GLUT4) at the sarcolemma. It is activated by IGF-1, insulin, and other growth factors. IL-6 is known to have a potentializing effect on an acute, short-term exposure such as an exercise-induced production of IL-6 by muscle cells [30] and an inhibiting effect on a chronic, long-term exposure such as an inflammatory state [31,32].

**Figure 6:**
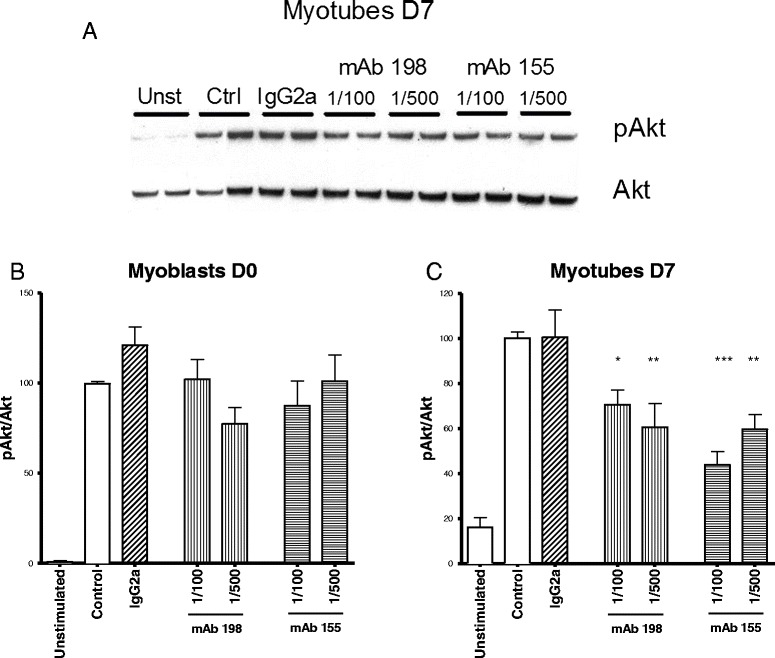
**Akt phosphorylation is decreased by AChR antibodies in myotubes but not in myoblasts.** Immortalized human muscle cells (LHCN) were differentiated over 7 days (D7) or kept as myoblasts (D0). AChR monoclonal antibodies (mAb 198 or mAb 155) or isotype control (IgG2a) were added 48 h before the analysis. The cells were stimulated with insulin or not (Unstimulated) and the proteins were extracted and analyzed via a Western Blot. A representative image of the Western Blot result is shown in panel **A**. The ratio between pAkt and total Akt band intensities is represented in graphs for myoblasts **(B)** and myotubes at D7 **(C)**. In each group, the sample size was n = 5–7 from three independent experiments. Asterisks represent significance given by an ANOVA analysis versus the control condition (Dunnett’s post test). Mean ± SEM is represented. ***p < 0.001; **p < 0.01; *p < 0.05.

We investigated the pAkt/total Akt ratio in response to insulin in human muscle cells exposed to AChR monoclonal antibodies over the course of 48 h, which corresponds to a long-term exposure in order to mimic the pathological myasthenic situation. In myoblasts, the antibodies did not change significantly the pAkt/Akt ratio (Figure 6B), but did so in myotubes (Figure 6C). As expected, Akt phosphorylation was reduced in the presence of anti-AChR antibodies.

These results clearly validate the involvement of the IGF-1/Akt pathway identified in the microarray and open a new field of investigation in the pathogenesis of myasthenia in muscles.

## Discussion

The aim of this study was to investigate the molecular mechanisms occurring in the muscle of MG patients and EAMG models. Our major findings are as follows: 1) The muscle signatures associated with the disease were strikingly similar in MG patients and in induced EAMG in rats, and revealed the involvement of IL-6 and IGF-1 pathways; 2) IL-6 had an altered expression in the muscles of EAMG models and MG muscle compared to controls, and was induced in cultured muscle cells treated with anti-AChR antibodies; 3) Akt, a downstream effector of IGF-1 pathway on which IL-6 is known to have a negative effect, exhibited defective phosphorylation in cultured muscle cells treated with anti-AChR antibodies.

### Role of IL-6 in pathogenic mechanisms in MG muscle

In this study, we have identified disease-associated gene signatures and pathways with a microarray approach. It is worth noting that the IL-6 and IGF-1 pathways were found to be significantly deregulated in both the spontaneous human disease and the model induced in rats.

IL-6 was originally discovered within the immune system. Numerous studies, however, have revealed that IL-6 is produced by, and released from, contracting skeletal muscles during exercise or in response to external and internal stress signals [27,33]. This release occurs in the absence of muscle damage. Tsujinaka et al. [34] have demonstrated that overexpression of IL-6 in transgenic mice causes muscle atrophy and increases levels of cathepsins in muscles, indicating that IL-6 is involved in regulating muscle protein breakdown, which can be prevented by the administration of anti-IL-6R antibodies [35]. Muscle atrophy is sometimes observed in MG patients, especially in type II fibers [36,37], but whether atrophy is due to the increased IL-6 production by the autoantibodies remains an open question. This possibility is in agreement with a study by Tuzun et al., who demonstrated a direct role for IL-6 in muscle cell destruction [38] and that of Aricha et al., who showed a significant improved clinical state of MG-induced rats after anti-IL-6 treatment [39].

Our *ex vivo* experiments show a decreased level of IL-6 mRNA in the muscle of EAMG rats, whereas the IL-6 mRNA level was increased in the muscle of EAMG mice. This apparent contradiction may be linked to the severity of the disease: muscles from rats were harvested when they reached an elevated score, whereas mice did not systematically show clinical signs. Moreover, the most affected mice had the lowest IL-6 mRNA levels, which suggests a correlation between the level of IL-6 and the degree of muscle damage. Thus, except in cases of very severe disease, IL-6 production appears to be activated in vitro (muscle cells stimulated with anti-AChR antibodies) and ex vivo (IL-6 protein in human MG muscle and IL-6 mRNA in mouse muscle). Interestingly in MG patients, the increase of IL-6 mRNA was not significant (data not shown), while the level of IL-6 protein was statistically increased. This could be explained by the higher stability of the protein IL-6 compared to the mRNA that is quite unstable [40]. The functional involvement of IL-6 was further demonstrated in the cultures of muscle cells treated by MG sera or anti-AChR monoclonal antibodies (mAb 198) that displayed a significant increase in IL-6 protein production.

Together, these data show that IL-6 is highly deregulated in the muscle of MG patients and EAMG models. The natural course of the disease may then have two stages, with different levels of IL-6 transcripts produced by the muscle: increased IL-6 production during mild stages, and decrease in severe stages. In order to better understand this process, it would be informative to investigate the muscle characteristics in those stages; myoatrophy, for example, may explain this change in the response. Although IL-6 high levels are known to promote muscle atrophy, whether IL-6 production is reduced in the atrophic fibers has not been described.

#### Why and how is IL-6 regulated in MG muscle?

The mechanisms underlying the increased production of IL-6 by the muscle cells after the attack by the anti-AChR antibodies are not clear. IL-6 is regulated by cytokines. TNF-alpha and IL-1beta induce IL-6 production by cultured skeletal muscle cells via the activation of a MAPK signaling pathway [41,42]; IL-10 has an inhibitory effect on IL-6 mRNA and protein expression. IL-6 production is also controlled by an autocrine regulation exerted by IL-6 [43]. Therefore, the increase in IL-6 induced by the antibodies could be mediated by increased TNF-alpha production. We tested this assumption by measuring TNF-alpha in the muscle after treatment with the mAb 198, but TNF-alpha was undetectable (data not shown). This result did not support this hypothesis, but IL-6 can potentially be modulated by many different cytokines and further investigations are needed to determine the mechanism responsible for this increase.

It is worth noting that SPMG sera had a weaker effect on IL-6 production than the SNMG sera, which does not support the hypothesis that the IL-6 increase was dependent on anti-AChR antibodies, although the subsequent experiments using monoclonal anti-AChR antibodies demonstrated that they were sufficient to induce IL-6 production by the muscle cells. This finding could be explained by the immediate effect of SPMG sera on the internalization of AChR, limiting this functional mechanism. In addition, anti-AChR antibodies in SPMG sera are degraded with their targets during the internalization process. It is therefore possible that anti-AChR antibodies become limiting in the *in vitro* assay, whereas they are produced continuously *in vivo*. In SNMG, the internalization induced by the sera is very limited [44], favoring this newly described mechanism. In accordance with this hypothesis, Leite et al. [45] show that some SNMG patients possess anti-AChR antibodies detectable only on whole clustered AChR. One can speculate that these anti-AChR antibodies against clustered-AChR do not reduce AChR numbers, but lead to a functional signaling effect. All SNMG were negative for anti-MuSK antibodies, but it is possible that some of them had anti-LRP4 antibodies that may have a similar effect. We can also suppose that other factors present in the SNMG sera are able to induce a similar response to the AChR antibodies. The precise mechanisms are still elusive but they appear specific to myasthenia since no control isotype antibodies or other muscle disease sera elicit this response from muscle cells.

### Consequences of the overproduction of IL-6 in MG muscle: a new mechanism of action of the anti-AChR antibodies?

Three mechanisms of action of anti-AChR antibodies have been described thus far: accelerated internalization of AChR, complement-dependent degradation of the receptor, and blocking of acetylcholine binding to AChR [46]. Our results provide arguments in favor of an additional mechanism of action that could be defined as a functional mechanism.

The increase in IL-6 production by muscle cells exposed to anti-AChR antibodies could have significant consequences on muscle biology, but also on immune responses. Indeed, myoblast proliferation is stimulated by IL-6, and satellite cell proliferation is regulated by an autocrine IL-6 effect [47,48]. Further evidence provided by genetic studies showed that IL-6-deficient mice are resistant to the development of autoimmune diseases such as myocarditis and EAMG [49,29]. This finding was associated with a significant reduction in germinal center formation, reduction in anti-AChR antibody production, and impaired upregulation of complement C3. Because anti-AChR antibodies and C3 activation contribute to the autoimmune destruction of AChR, therapeutic downregulation of IL-6 could control the deleterious events occurring at the neuromuscular junction in EAMG and likely in the early stages of MG [29]. Interestingly, the IL-1 receptor-mediated therapeutic effect in the murine EAMG model is associated with downregulation of TNF-alpha, IL-6, and C3 [50]. In addition, we very recently showed that anti-IL-6 therapy improves the clinical state of MG [39]. The increased IL-6 production induced by some antibodies could in turn lead, in addition to its pro-inflammatory role, to several functional consequences affecting the metabolism of the muscle, its differentiation, and its regeneration.

### Is there a link between IL-6 overproduction and the IGF-1/Akt pathway in MG?

We have shown that monoclonal anti-AChR antibodies reduce Akt phosphorylation in response to insulin. Interestingly, mAb 155 displayed a dose-dependent response and mAb 198 did not; the latter had a stronger effect on IL-6 production. Several explanations could be proposed: 1) There is a threshold effect of IL-6 on Akt phosphorylation that was exceeded in both mAb 198 dilutions, but only in one of the mAb 155 dilution; 2) the effect is not dependent on IL-6; 3) we cannot exclude that a low level of IL-6 (in the case of Mab155) is due to its overconsumption by the cells.

Although it has been described that IL-6 has an inhibiting effect on Akt pathway in chronic exposure [31], it remains to be determined if the action of anti-AChR antibodies is mediated by IL-6. Unveiling impaired Akt phosphorylation caused in muscle cells by AChR antibodies is a first step to defining those consequences. It remains to be determined how this mechanism is translated in terms of muscle physiology, since Akt is involved in many processes such as growth, glucose metabolism, and so on. It is interesting that differentiated and undifferentiated muscle cells do not respond in the same way to anti-AChR antibodies, indicating that each cell type may play a distinct role in pathogenesis. Although not frequent, atrophy may be observed in MG and could be a direct consequence of muscle growth impairment by Akt pathways [36]. Metabolic aspects should also be investigated since they may play a role in muscle weakness and fatigue, in which case a complementary therapy activating this metabolic pathway could alleviate myasthenia symptoms.

## Conclusions

Our results describe a new mechanism of action of the anti-AChR antibodies in the muscle of MG patients and EAMG models, including IL-6 and IGF-1/Akt pathways. This mechanism could be defined as a functional mechanism. The reduced phosphorylation of Akt by the anti-AChR antibodies may have a significant impact on the muscle fatigability observed in MG patients. The increased production of IL-6 by MG muscle may have an effect on the muscle function, and on systemic inflammation. Modulating their protein products could represent a new therapeutic strategy for MG.

## Additional file

Additional file 1: Table S1. Deregulated genes for the IGF-I and IL-6 pathways. Among the genes described in the IGF-I pathway, 20 were deregulated in MG patients, and 12 in EAMG rats. Concerning the IL-6 pathway, 13 genes were deregulated in patients and 13 in rats. Highlighted genes are common between the two pathways.

## Abbreviations

MG: Myasthenia Gravis
EAMG: experimental autoimmune MG
SPMG: Seropositive MG
SNMG: Seronegative MG
AChR: AcetylCholine Receptor
MuSK: Muscle Specific Kinase
LRP4: Low-density lipoprotein Receptor-related Protein 4
IL-6: interleukin-6
IGF-1: Insulin-like Growth Factor-1
Akt: v-akt murine thymoma viral oncogene homolog
