# Supplementary material for: IL-6 and Akt are involved in muscular pathogenesis in myasthenia gravis
Source: Acta Neuropathol Commun. 2015 Jan 15;3:1. doi: 10.1186/s40478-014-0179-6 (PMC4308930; doi:10.1186/s40478-014-0179-6)
Supplement: Additional file 1: Table S1. — Deregulated genes for the IGF-I and IL-6 pathways. Among the genes described in the IGF-I pathway, 20 were deregulated in MG patients, and 12 in EAMG rats. Concerning the IL-6 pathway, 13 genes were deregulated in patients and 13 in rats. Highlighted genes are common between the two pathways. [file 40478_2014_179_MOESM1_ESM.docx]

**Additional file 1: Table S1: Deregulated genes for the IGF-I and IL-6 pathway**

Among the genes described in the IGF-I pathway, 20 were deregulated in MG patients, and 12 in EAMG rats. Concerning the IL-6 pathway, 13 genes were deregulated in patient, 13 in rats. Highlighted genes are common between the two pathways.

| IGF-1 pathway | |  |
| --- | --- | --- |
|  |  |  |
|  | Human transcriptome | Rat transcriptome |
| Up | JUN | Eif4ebp1 |
|  | XBP1 | NfkbIa |
|  | MAP2K4 |  |
|  | IGF1R |  |
|  | MAP4K4 |  |
|  | REG1P |  |
|  | SOS2 |  |
|  | SLC2A4 |  |
|  | PRKCQ |  |
|  | RPS6KA3 |  |
|  | GRB2 |  |
|  | EGR1 |  |
|  | PFKL |  |
|  | FOS |  |
|  | PIK3R1 |  |
|  | BTK |  |
| Down | MAP4K5 | Egr1 |
|  | RPS6KA2 | Fos |
|  | INPPL1 | Rrad |
|  | CASP9 | Hras |
|  |  | Rps6kb1 |
|  |  | Map2k6 |
|  |  | Raf1 |
|  |  | Jun |
|  |  | Ccnd1 |
|  |  | Fkbp1a |
|  |  |  |
|  |  |  |
| IL-6 pathway | |  |
|  |  |  |
|  | Human transcriptome | Rat transcriptome |
| Up | JUNB | Il6ra |
|  | MAP2K4 | Crp |
|  | IL6ST | Jak2 |
|  | GRB2 | Cebpd |
|  | PIK3R1 | Cebpb |
|  | TNFSF10 | NfkbIa |
|  | JUN |  |
|  | NR3C1 |  |
|  | FOS |  |
|  | SOS1 |  |
|  | SOS2 |  |
| Down | A2M | Rps6kb1 |
|  | TIMP1 | Met |
|  |  | Ccl2 |
|  |  | Jun |
|  |  | Raf1 |
|  |  | Fos |
|  |  | Hras |
